# Formulation of silver phosphate/graphene/silica nanocomposite for enhancing the photocatalytic degradation of trypan blue dye in aqueous solution

**DOI:** 10.1038/s41598-024-66054-5

**Published:** 2024-07-10

**Authors:** M. S. Showman, R. Y. Omara, E-S. Z. El-Ashtoukhy, H. A. Farag, M. M. Abd El-Latif

**Affiliations:** 1 Fabrication Technology Department, Advanced Technology and New Materials Institute, City of Scientific Research and Technological Applications, Alexandria, Egypt; 2 Chemical Engineering Department, Faculty of Engineering, Alexandria University, Alexandria, Egypt

**Keywords:** Silver phosphate, Photocatalysis process, Silver nanocomposites, Trypan blue dye, Environmental sciences, Chemistry, Materials science

## Abstract

Photocatalytic degradation of several harmful organic compounds has been presented as a potential approach to detoxify water in recent decades. Trypan Blue (TB) is an acidic azo dye used to distinguish live cells from dead ones and it's classified as a carcinogenic dye. In this study, silver phosphate (Ag_3_PO_4_) nanoparticles and novel Ag_3_PO_4_/graphene/SiO_2_ nanocomposite have been successfully prepared via simple precipitation method. Afterward, their physical properties, chemical composition, and morphology have been characterized using SEM, EDS, TEM, SAED, BET, XRD, FTIR and UV–VIS spectroscopy. The specific surface area of Ag_3_PO_4_ and Ag_3_PO_4_/G/SiO_2_ nanocomposite were reported to be 1.53 and 84.97 m^2^/g, respectively. The band gap energy of Ag_3_PO_4_ and Ag_3_PO_4_/G/SiO_2_ nanocomposite was measured to be 2.4 and 2.307 eV, respectively. Photocatalytic degradation of Trypan blue (TB) was studied at different parameters such as pH, catalyst dosage, initial concentration, and contact time. The results showed that, at initial dye concentration of 20 ppm, pH = 2, and using 0.03 g of Ag_3_PO_4_/G/SiO_2_ as a photocatalyst, the degradation percent of TB dye in the aqueous solution was 98.7% within 10 min of light exposure. Several adsorption isotherms such as Langmuir, Freundlich, and Temkin adsorption isotherms have been tested in addition to the photocatalytic degradation kinetics. Both catalysts were found to follow the Langmuir isotherm model and pseudo-second-order kinetic model. Finally, the possible photocatalytic performance mechanism of Ag_3_PO_4_/G/SiO_2_ was proposed.

## Introduction

Organic pollutants are considered one of the main pollutants in water, with a wide range of toxicity. Dyes, plant and animal medications, and petroleum organic pollutants are among the organic pollutants that have posed a serious threat to mankind and aquatic species^1^. There are currently over 10,000 commercially available dyes, and over 700,000 tons of dyes are produced each year^1,2^. Trypan blue is an artificial dye that belongs to the azo dye family and is generally used in various industries, including textiles, prescription drugs, and biomedical studies. Its colorful blue shade makes it broadly employed as a staining agent in biological and clinical laboratories^3^. However, the huge use of trypan blue has brought about its presence in business effluents and wastewater. Due to insufficient treatment techniques in positive commercial tactics, trypan blue, like many other synthetic dyes, unearths its way into wastewater streams^4^. Disposal of untreated or inadequately treated wastewater containing trypan blue can result in the infection of water in our bodies, posing a hazard to aquatic ecosystems and probably affecting human health. The staying power of those dyes inside the environment is a motive for difficulty, as they could resist conventional water treatment techniques and acquire over time^2^. The environmental impact of trypan blue and other synthetic dyes stems from their capacity for toxicity, mutagenicity, and carcinogenicity. When launched into natural water systems, those dyes can disrupt the balance of aquatic ecosystems, damage aquatic life, and negatively impact biodiversity. Moreover, their presence in our bodies might also lead to the formation of unwanted byproducts, exacerbating the environmental impact^4^. As a result, a plethora of technological remedies have been developed to remove organic pollutants such as dyes, including physical methods such as adsorption and biological degradation and chemical techniques such as ozonation and chlorination, in addition to the advanced oxidation process (AOP)^4–6^. Amongst AOP techniques, one well-known and efficient method for totally decomposing organic molecules found in contaminated wastewater is photocatalytic degradation^5^. Using the right photocatalyst and energy from light, this process produces extremely reactive hydroxyl (OH^·^) radicals. These radicals have the ability to change harmful substances found in water into more harmless byproducts, including CO_2_, H_2_O, and other inorganic ions^7,8^. The safety of both people and the environment is guaranteed by this change. When compared to other traditional techniques, photocatalytic degradation has several advantages. It is a straightforward, efficient instrumental technique that has nonselective oxidation and simple, controllable processes. Furthermore, it can completely mineralize and degrade synthetic organic dyes and is reasonably priced^9^. A semiconductor photocatalyst that is activated by absorbing photons is necessary for this process to occur. Interestingly, the photocatalyst can speed up the reaction without being consumed^10^. Metal nanoparticles are the most commonly utilized photocatalysts, and their characteristics are closely related to particle shape, size, geometry, and morphology^11^. Nanoparticles are tiny grains with diameters less than 100 nm that have sparked substantial interest due to their distinct chemical and physical properties. They have shown potential applications in a variety of sectors, including medicine, solar cells, and nano-devices^12–14^. The photodegradation of organic dyes is running according to a commonly suggested mechanism. First step (Eq. 1) involves light absorption by the semiconducting material, when the emitted photons have energy (${\text{h}}\nu$) at least equal to or higher than the semiconductor energy band gap (*E*_g_); electrons in the valence band will be stimulated and excited when illuminated by light. The excess energy of this excited electrons will promote the electrons to the conduction band creating negative electrons (e^−^), and positive holes (h^+^) will be formed in the valence band. In the second step, (Eq. 2) the positive holes will react with the water molecules to form hydrogen gas and hydroxyl radicals (OH^·^), while the negative electrons will react with oxygen molecules (Eq. 3) to form superoxide anions (O_2_^·−^)^15–21^. Equation 4 shows that the organic matters will be degraded via successive attack by OH^·^, h^+^, and O_2_^·−^.

1 $$ {\text{Photocatalyst}} + {\text{h}}\nu = {\text{e}}^{ - } + {\text{h}}^{ + } \quad \quad \quad \left( {{\text{Photons}}\;{\text{absorption}}} \right) $$

2 $$ {\text{h}}^{ + } {\text{ + H}}_{{2}} {\text{O}} = {\text{OH}}^{ \cdot } \quad \quad \quad \quad \quad \left( {{\text{Oxidation}}} \right) $$

3 $$ {\text{e}}^{ - } + {\text{O}}_{{2}} = {\text{O}}_{{2}}^{ \cdot - } \quad \quad \quad \quad \quad \left( {{\text{Reduction}}} \right) $$

4 $$ {\text{h}}^{ + } ,\;{\text{OH}}^{ \cdot } ,\;{\text{O}}_{{2}}^{ \cdot - } + {\text{TB}} = {\text{CO}}_{{2}} + {\text{H}}_{{2}} {\text{O}}\quad \quad \quad \left( {{\text{Degradation}}} \right) $$

It is expected that by using graphene/SiO_2_ nanocomposite the photocatalytic activity of Ag_3_PO_4_ would be improved, as the semiconducting nanocomposite would trap the excited electrons in the conduction band, as a consequence, the possibility of recombination with the valence band’s holes will be diminished.

**Figure 1:**
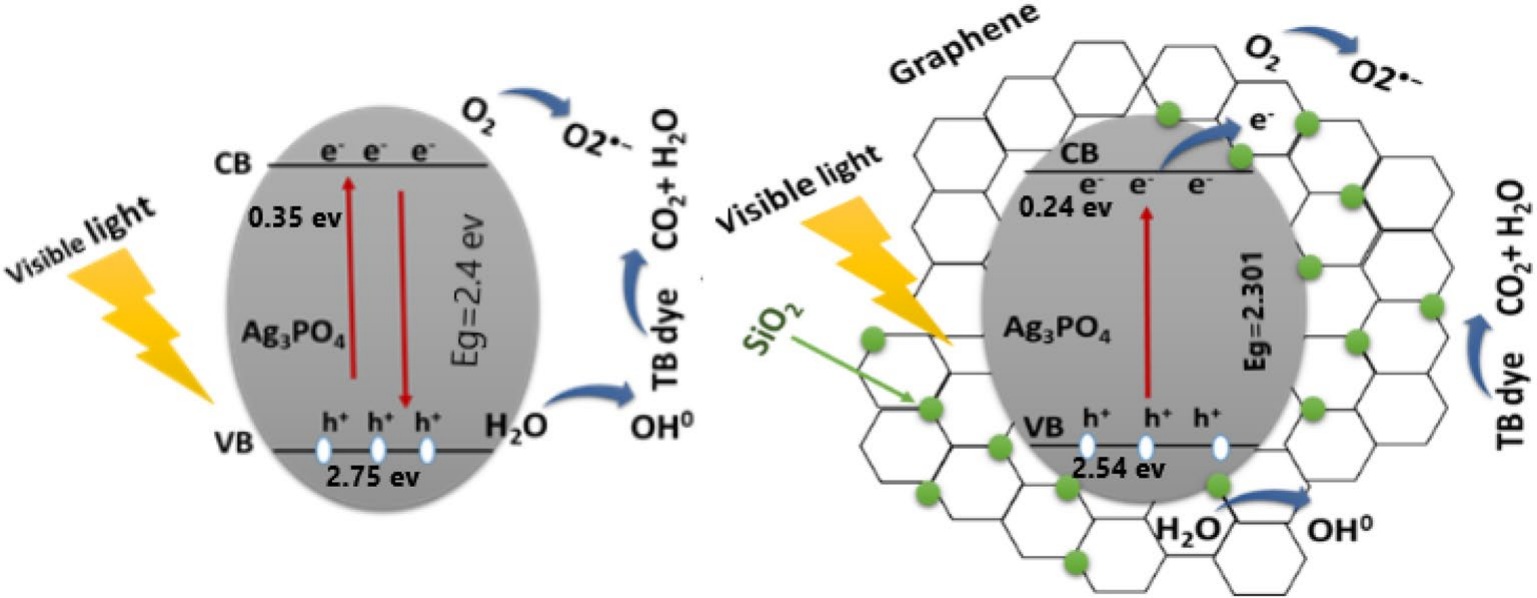
Photocatalytic mechanism of Ag_3_PO_4_, and Ag_3_PO_4_/G/SiO_2_ Composite.

Many researchers have proved that Ag_3_PO_4_ has a sensitivity toward visible light as shown in Fig. 1, therefore, it exhibited high photocatalytic performance for organic dyes decomposition^22^. Although the outstanding photooxidation capability has been proved through many researches, there are many obstacles that hinder its wide and practical application; the main one is the photo corrosion^23^. Photo corrosion occurred when the photo-generated electrons under light irradiation are absorbed by the silver ions (Ag^+^) and consequently reduced to metallic silver (Ag^0^), which would deposit on the surface of Ag_3_PO_4_. As a result, the structure of Ag_3_PO_4_ could be destroyed, and thus the photocatalytic activity would decrease^24,25^. In order to overcome this barrier, numerous studies tend to couple different semiconductors together seeking to improve the separation and transportation of photo-generated charges^26–29^. For instance, Yunyun et al.^30^, prepared Ag_3_PO_4_/RGO/Bi_2_WO_6_ nanocomposite and tested its efficiency on the degradation of tetracycline dye (TC) (20 mg/L), the percent of removal was 90% within 90 min. 50 wt% Ag_3_PO_4_/GO/g-C_3_N_4_ was synthesized by JiaYan^31^ using the chemical precipitation method to degrade Rh (20 mg/ L) where the removal was 94.8%. And Zhou^32^ presented Ag_3_PO_4_/GO/NiFe_2_O_4_ using ion-exchange deposition to remove RhB (10 mg/L) within 15 min, the degradation was 96%. The aim of this study is to improve the efficiency of the organic TB dye degradation by the generation of silver phosphate nanoparticles and novel Ag_3_PO_4_/G/SiO_2_ nanocomposite as photocatalysts using the simple chemical precipitation method. The shape, structure and elemental composition of this photocatalyst are carefully studied using SEM, EDS, TEM, SAED, BET surface area, XRD, FTIR and UV–VIS (VIS) spectroscopy. The focus on these elements is to improve the design and composition of the catalyst for optimal efficiency. The scope of the study includes the following objectives: The primary objective is to form a low-cost, high-efficiency photocatalyst for the breakdown of TB dye. By focusing on the degradation of a widely used dye with various applications, the work addresses a real-world contamination issue. Second, the study examines parameters that influence degradation efficiency, such as duration, medium pH, photocatalyst dosage, and dye concentration.

Furthermore, the adsorption behavior of the catalysts has been studied to understand the nature of the process, which significantly influences the photocatalytic degradation process. By thoroughly investigating these parameters, the project aims to provide insights into the best circumstances for dye degradation, thereby improving our ability to prevent water pollution.

## Experimental part

### Materials

Silver nitrate (AgNO_3_) of purity 99.9% and Ammonium dihydrogen phosphate salt NH_4_ (H_2_PO_4_) (99% purity) were purchased from TECHNO PHARMCHEM (INDIA). Trypan Blue (C_34_H_28_N_6_O_14_S_4_) anionic dye (Mwt = 960.8, purity 70%) and Polyvinylpyrrolidone (PVP) were obtained from Sigma Aldrich. For pH adjustment: Sodium hydroxide and citric acid solutions were used (0.1M). Distilled water was used to prepare the different dye solutions. Rice husk (RH) was obtained from the local market that collected from the farmlands near to Alexandria, Egypt.

### Synthesis of Ag_3_PO_4_

At room temperature and in the absence of light, Ag_3_PO_4_ nanoparticles were prepared using a simple precipitation method by dissolving 4.26 g of AgNO_3_ in 200 ml distilled water and stirred for 1 h (solution 1). 1.2 g of NH_4_ (H_2_PO_4_) were added to 200 ml distilled water and stirred for 30 min (solution 2). The later was added dropwise to (solution 1) under continuous stirring at 300 rpm for 2 h then the precipitate was formed at pH 5. Finally, the precipitate was separated using centrifugation at 6000 rpm and washed several times with distilled water and ethanol in ratio (1:1), then placed in a vacuum oven overnight at 70 °C^23,33^.

### Synthesis of Ag_3_PO_4_/G/SiO_2_ nanocomposite

A graphene/SiO_2_ nanocomposite from rice husk (RH) was previously synthesized as follows: in a one-step green manner using chemical activation, RH was physically prepared (sieved, washed many times with distilled water, dried in an oven at 105 °C for 24 h, then ground). after that, the husk was chemically activated with KOH at 850 °C for 2 h in a muffle furnace. Finally, the husk was rinsed with distilled water to remove any residual KOH and dried at 100 °C for 24 h^34^. To prepare the silver phosphate graphene/silica nanocomposite (Ag_3_PO_4_/G/SiO_2_), first 0.2 g from the as prepared (Graphene/SiO_2_) nanocomposite was dispersed in 100 ml of distilled water and sonicated for 30 min, then 100 ml of AgNO_3_ solution (0.125 M) was added to the dispersion and stirred for an hour, after that 5 g of PVP were added and vigorously stirred for another hour. 0.6 g of NH_4_ (H_2_PO_4_) was dissolved in 100 ml of distilled water and stirred for 30 min before being added dropwise to the prepared mixture to reach pH 7 and left for 1 h under continuous stirring overnight. The precipitate was separated by centrifugation and washed several times with water and ethanol and dried using a vacuum oven at 70 °C.

### Catalysts characterization

In order to investigate the functional groups on the surface of the as-prepared catalysts, Fourier transform infrared spectra (FTIR, 8400 S Shimadzu, Japan) of the samples were collected in the range of 400–4000 cm^−1^. X-ray diffraction (XRD 7000 Shimadzu, Japan) analysis was conducted to identify the structure crystallinity. The surface morphology of the was investigated using the scanning electron microscopy (SEM, JEOL JSM 6360LA, Japan). Energy dispersive x-ray spectroscopy analysis (EDS) with mapping were performed to study the chemical composition. Transmission electron microscopy (TEM, JEOL JEM 100CX, Japan) was used to characterize the structure and particle size of the samples. The identification of the degree of crystallinity of the prepared samples have also been observed using selected area electron diffraction (SAED) patterns. The surface area was estimated by Brunauer–Emmett–Teller technique (BELSORP-mini X (S/N: 149, Version 1.0.9.0 Instrument, Japan). The optical property was examined by using a UV–visible spectrophotometer (PG Instrument, Model: T60UV, UK).

### Photocatalytic degradation of TB dye

The photocatalytic degradation behavior of TB (anionic dye) using Ag_3_PO_4_ catalyst and Ag_3_PO_4_/G/SiO_2_ nanocomposite under visible light illumination was investigated. The photocatalytic degradation reaction was conducted in a simple photocatalytic reactor which simply consists of 500 ml glass beaker, magnetic stirrer and a 100-Watt tungsten lamp as a light source of wave length ranged from 320 to 2400 nm. In a single experiment, the mixture of catalyst and TB dye solution was magnetically stirred in the dark for 30 min to establish an adsorption–desorption equilibrium of TB dye on the surface of catalysts before irradiation. In the absence of catalyst and under visible light irradiation, the degradation of the dye was almost negligible. Different parameters have been studied in order to identify the best condition for the photocatalytic degradation of the dye. The parameters which have been studied are the initial dye concentration (20, 30, 40, 50, and 100 ppm), pH of the dye solution (2, 4, 6, 8, and 10), and catalyst dosage (0.01, 0.02, 0.03, 0.05 g). Moreover, the zero-point charge (pHzpc) of Ag_3_PO_4_ and the composite was detected. Different doses from each photocatalyst were added to a 100 ml of the dye solution and was magnetically stirred firstly in dark for 30 min to reach equilibrated adsorption, then the lamp was illuminated. Samples are taken at the given time intervals (2, 4, 6, 8, 10, 15 min). Nanoparticles were separated from the suspension by centrifugation at 6000 rpm for 10 min. The concentrations of TB were analyzed at 590 nm, using a UV–Vis spectrophotometer. The percentage of dye degradation is calculated from the following formula^25,34^:

5 $$ {\text{\% Dye}}\;{\text{degradation }} = { }\frac{{C_{0 - } C}}{{C_{0} }} \times 100 $$

where; C_0_ and C are the initial dye concentration and concentration of the dye at time t in (mg/l), respectively.

## Results and discussion

### Physical and chemical properties of the prepared catalysts

**Figure 2:**
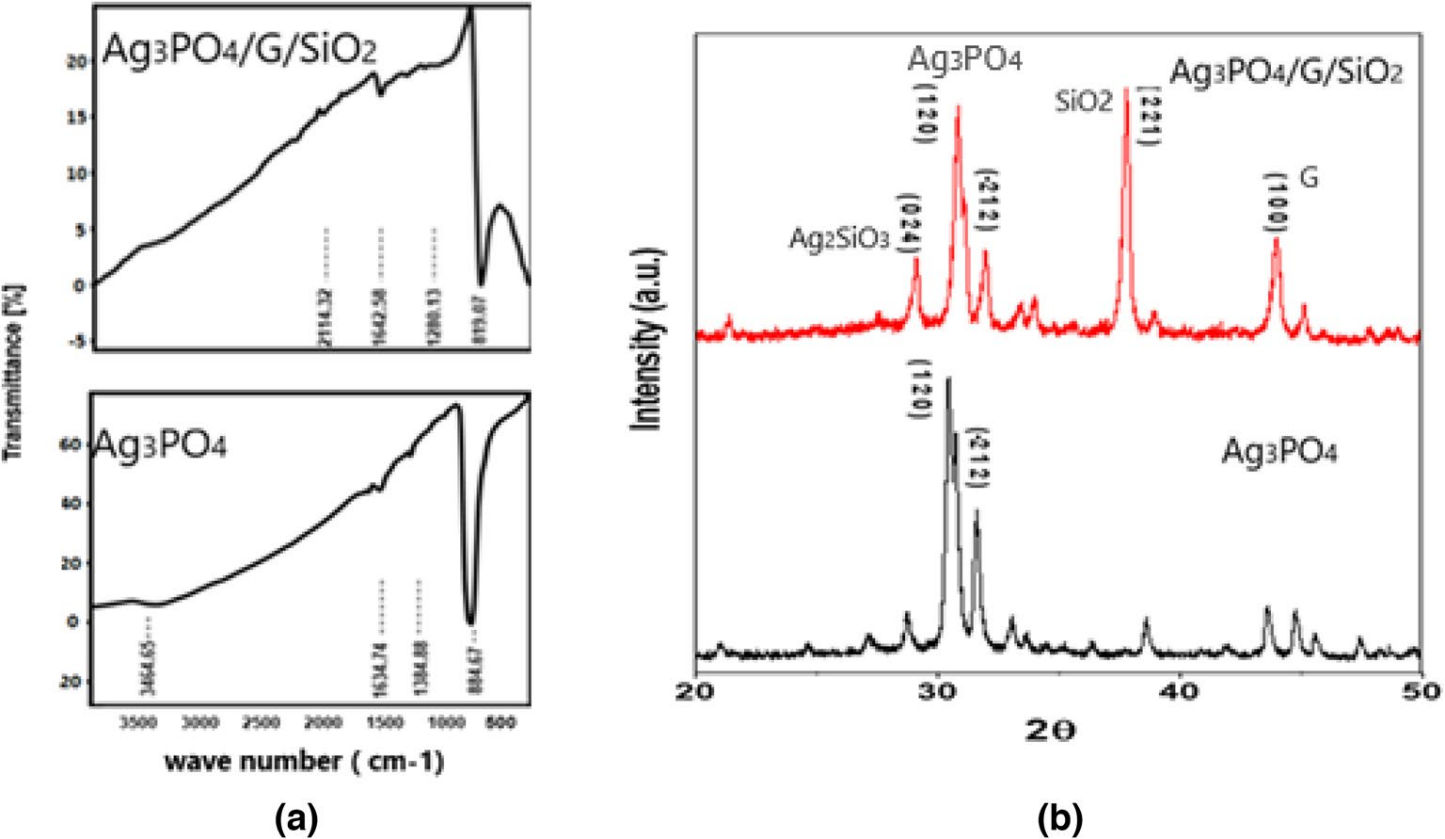
(**a**) FTIR and (**b**) XRD of Ag_3_PO_4_ and Ag_3_PO_4_/G/SiO_2_ composite.

FTIR analysis was used to identify the characteristic functional groups of the obtained samples. In Fig. 2a, Ag_3_PO_4_ showed a sharp beak at 884.67 cm^−1^ which is assigned to the vibration mode of (PO_4_^–3^) group. The small peak at 1384.88 cm^−1^ confirms the presence of residual water molecules because of the (OH^−^) vibration bond. The stretching modes at 3464.65 cm^−1^ and 1634.74 cm^−1^ are caused by the OH– defects^23,33^. FTIR analysis of Ag_3_PO_4_/G/SiO_2_ nanocomposite manifested a bond of C–Si at 2114.32 cm^−1^, and bending vibration of OH^−^ at 1642.58 cm^−1^. The absorption band at 1280.13 cm^−1^ is corresponding to O–Si–O bond^23,33,34^.

Figure 2b shows the XRD patterns of the of Ag_3_PO_4_ and Ag_3_PO_4_/graphene/SiO_2_ which revealed a high crystallinity of both samples. The diffraction peaks at the 2θ values 30.500 and 31.680 corresponding to the reflections of (1 2 0) and (− 2 1 2) represent the Ag_3_PO_4_ monoclinic phase structure in both catalysts and agreed with (PDF 72–0122). For the composite a sharp peak observed at plane (2 2 1) which incident for nano silica crystals (PDF 85–0621), and the lattice plane (1 0 0) reveals formation of graphene sheets^34^.

**Figure 3:**
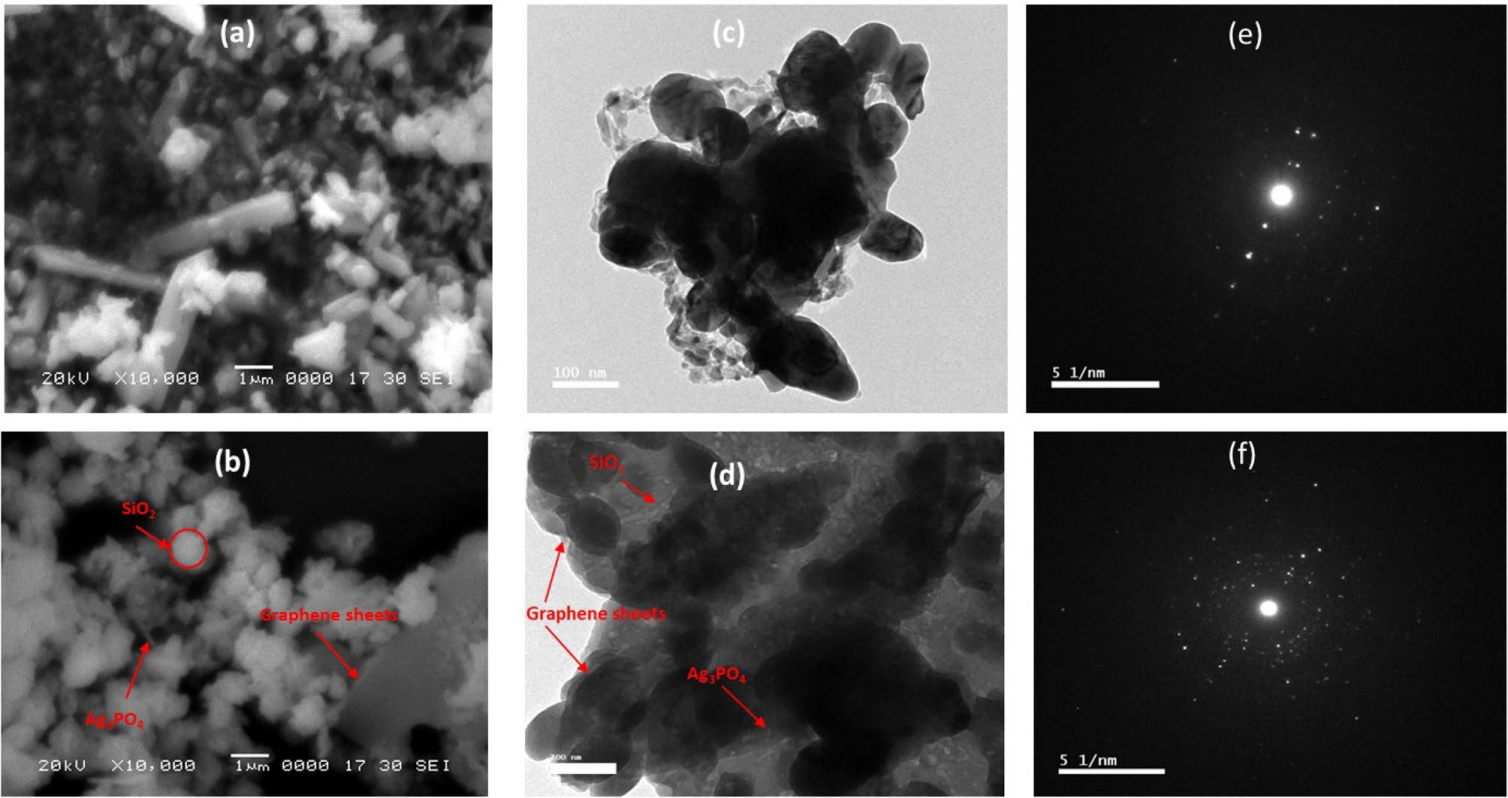
(**a** and **b**) SEM of Ag_3_PO_4_ and Ag_3_PO_4_/G/SiO_2_ composite. (**c** and **d**) TEM of Ag_3_PO_4_ and Ag_3_PO_4_/G/SiO_2_ composite. (**e** and **f**) SAED patterns of Ag_3_PO_4_ and Ag_3_PO_4_/G/SiO_2_ composite.

The formed peak at 2θ of 28.980 indicates the formation of monoclinic phase of silver silicate with (0 2 4) plane (PDF 85–0281), which stresses the attachment of Ag_3_PO_4_ particles with graphene/SiO_2_ composite through the nucleation on the PVP polymer chain^33^. All observed XRD miller indices were indexed in Table 1. The morphological structure of Ag_3_PO_4_ nanoparticles and Ag_3_PO_4_/G/SiO_2_ nanocomposite were investigated by SEM micrographs and the results are shown in Fig. 3a,b. It can be observed that the particles of Ag_3_PO_4_ consist of irregular agglomerated rods and spheres. Moreover, the images showed a lack of symmetry and uniformity in distribution and shape as presented in Fig. 3a^35^. The micrographs of Ag_3_PO_4_/G/SiO_2_ exhibited small particles of silica attached to the graphene surface with spheres and rods of Ag_3_PO_4_ (Fig. 3b). TEM images of the prepared samples showed that the rods and spheres of the silver phosphate are formed in random distribution (Fig. 3c). Whereas, in Fig. 3d, the images showed a semitransparent layer of graphene attached on its surface nano-spheres of silica distributed in random arrangement and rods and spheres of silver phosphate^33–35^. Figure 3e,f show SAED pattern of prepared Ag_3_PO_4_ and Ag_3_PO_4_/G/SiO_2_ composite in which the bright spots correspond to the cubic Ag_3_PO_4_ phase were appeared and confirm the well crystallinity of both catalysts.

**Table 1:** Miller indices (h k l) of observed XRD peaks.

Element	2θ (degree)	h	k	l
Graphene (G)	43.3	1	0	0
Silica (SiO_2_)	38	2	2	1
Silver phosphate (Ag_3_PO_4_)	31.68	− 2	1	2
Silver phosphate (Ag_3_PO_4_)	30.5	1	2	0
Silver silicate (Ag_2_SiO_3_)	28.9	0	2	4

**Figure 4:**
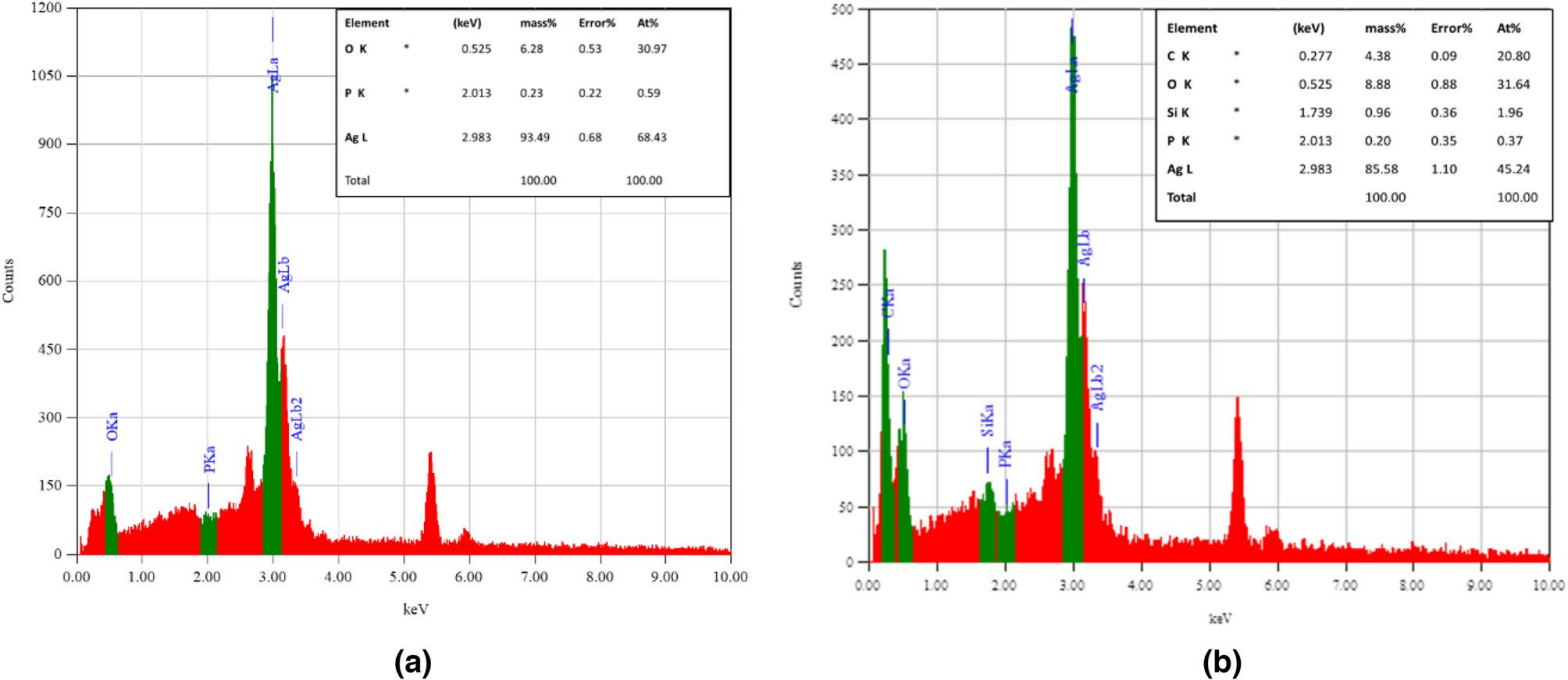
EDS elemental analysis of Ag_3_PO_4_ (**a**) and Ag_3_PO_4_/G/SiO_2_ composite (**b**).

Figure 4 shows the variation of EDS elemental analysis for the photocatalysts compositions. For Ag_3_PO_4_ (Fig. 4a), EDS analysis shows that Ag percentage is about 82%, P percentage of 10% and 5% of oxygen atoms. While for Ag_3_PO_4_/G/SiO_2_ composite (Fig. 4b) the percentages of silver and phosphorous will decreases to 62 and 3% respectively, after compositing with graphene/silica. Otherwise, the oxygen percentage increases to about 17% and the carbon percentage was nearly to 8% with a 2% silicon. These analyses are compatible with XRD analysis.

**Figure 5:**
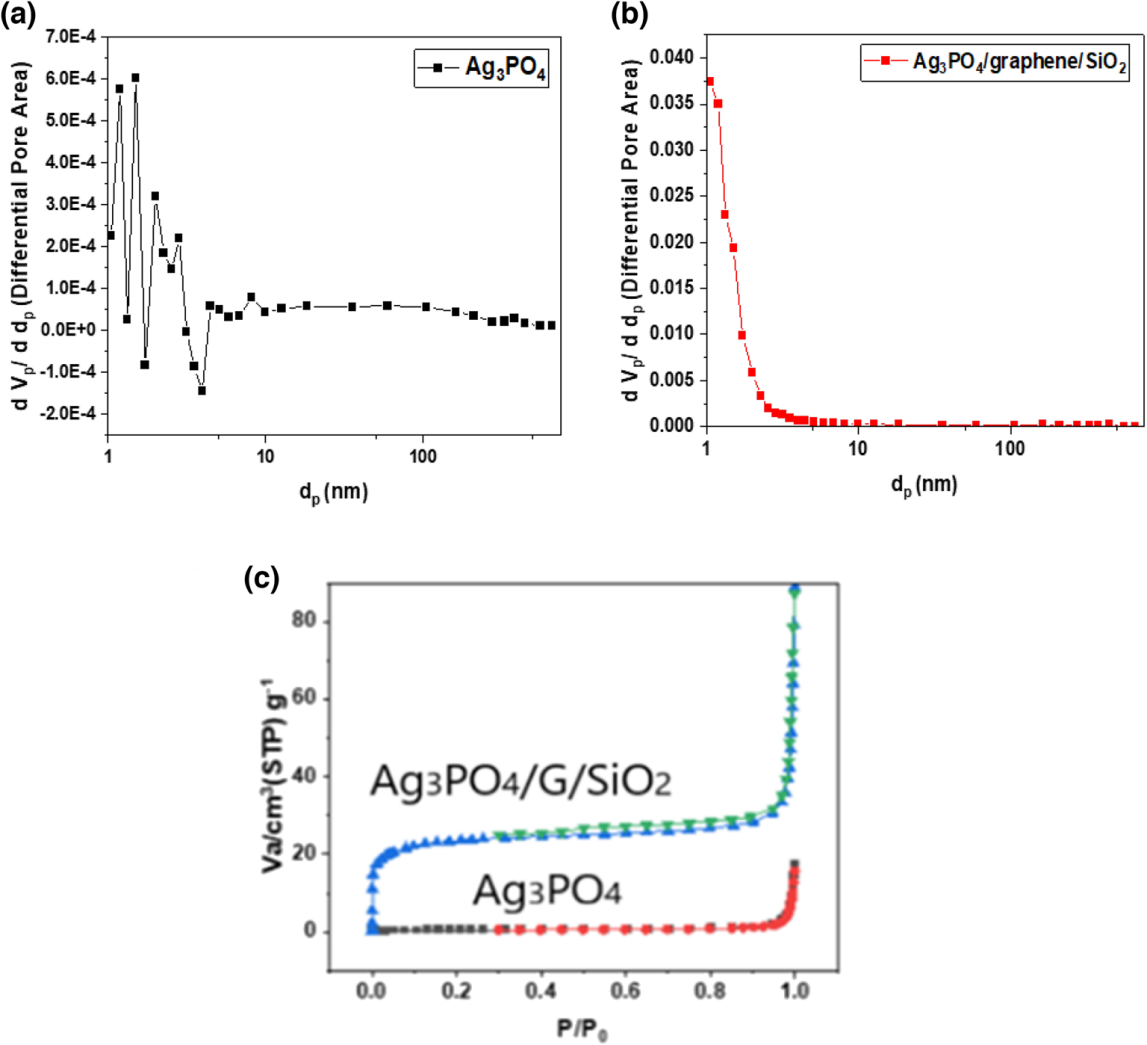
(**a**) BJH plot of Ag_3_PO_4_, (**b**) BJH plot of Ag_3_PO_4_/G/SiO_2_ (**c**) N_2_ Adsorption–Desorption isotherm on the surface of Ag_3_PO_4_ and Ag_3_PO_4_/G/SiO_2_.

Specific surface area of the as-synthesized photocatalysts was estimated by N_2_ adsorption technique and the results analyzed using BET theory. BJH analyses was performed to measure the average pore diameter of both catalysts and showed in Fig. 5a,b, where the average pore diameter of the composite was 5.4 nm, while for Ag_3_PO_4_ was 44.28 nm. Figure 5c showed that the Ag_3_PO_4_/G/SiO_2_ nanocomposite has large specific surface area which about 84.97 m^2^/g compared with 1.53 m^2^/g for Ag_3_PO_4_, the increase in specific area may be due to the incorporation of the highly surface area graphene/SiO_2_ composite which was previously prepared by Amr et al.^33^. Table 2 summarize the BET surface area analysis, it is observed that the Ag_3_PO_4_/G/SiO_2_ nanocomposite has smaller pores and also has a larger specific surface area per one gram, which indicates the presence of a large number of pores, i.e., its porosity^34^, which means the nanocomposite has greater ability for adsorption as mentioned before.

**Table 2:** Summarization of BET analysis of Ag_3_PO_4_ and Ag_3_PO_4_/G/SiO_2_.

Samples	S _BET_ (sq. m/g)	Total pore volume (V pore) (cm^3^·g^−1^)	Average pore diameter (nm)
Ag_3_PO_4_	1.53	1.0543E-02	27.496
Ag_3_PO_4_/G/SiO_2_	84.97	7.4674E-02	3.5152

**Figure 6:**
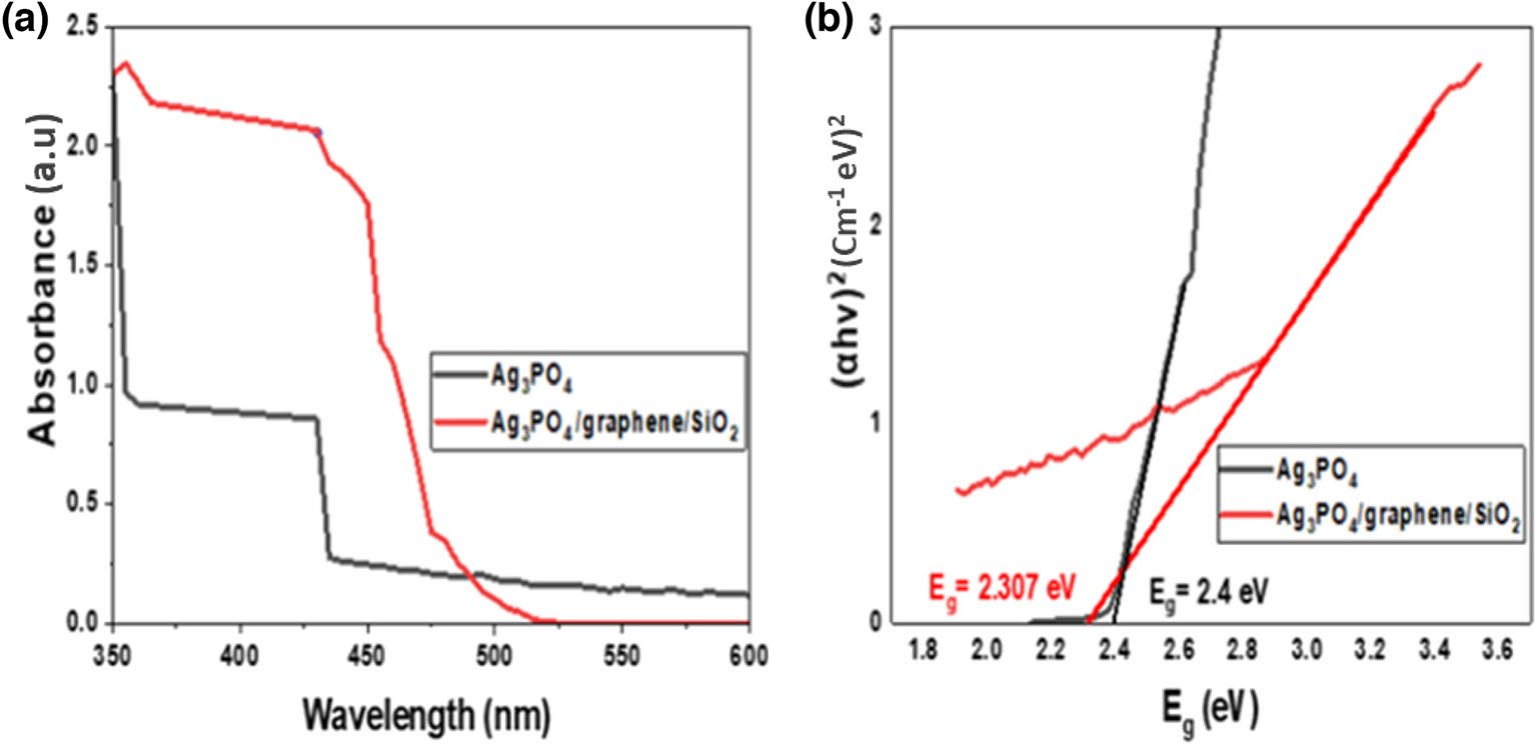
UV–VIS spectra of the Ag_3_PO_4_ and Ag_3_PO_4_/G/SiO_2_ (**a**), Tauc relationships of the prepared samples (**b**).

In order to study the optical behavior of Ag_3_PO_4_ and Ag_3_PO_4_/G/SiO_2_, UV/VIS DRS have been performed. As reported before, Ag_3_PO_4_ nanoparticles have a great sensitivity to visible light. The results showed that both as-prepared catalysts can absorb visible light, however, the nanocomposite revealed higher absorption capability as showed in Fig. 6a. This can be explained via the high conductivity of graphene which could act as an electron acceptor which in role enhance the rate of formation of positive holes on the composite surface and ensure the continuity of the photocatalytic performance^25,33,36^. Tauc equation^25,36^ has been used to determine the band gap energy of Ag_3_PO_4_ and Ag_3_PO_4_/G/SiO_2_ sample, according to the following formula.

6 $$ \alpha h\nu = {\rm A}\left( {h{\varvec{\nu}} - {\text{E}}_{g} } \right) ^{{{\raise0.7ex\hbox{$n$} \!\mathord{\left/ {\vphantom {n 2}}\right.\kern-0pt} \!\lower0.7ex\hbox{$2$}}}} $$

where E_g_, α, h, ${\varvec{\nu}}$, and A, are band gap energy, absorption coefficient, Planck constant, photon frequency, constant, respectively. Figure 6b showed the plot of $(\alpha {\text{h}}\nu )^{2}$ (indirect transition) versus energy band (${\text{h}}\nu$)^35^. According to the plot the band gap energy of Ag_3_PO_4_ was 2.4 eV and for the composite was 2.307 eV; hence, the obtained results enhance the strongest photocatalytic property of the composite.

### Studying the effect of operational parameters on the photocatalytic degradation efficiency

In order to study photocatalytic activity at different parameters, all the experiments were conducted first in dark medium for 30 min before exposure to the visible light.

#### pH effect

**Figure 7:**
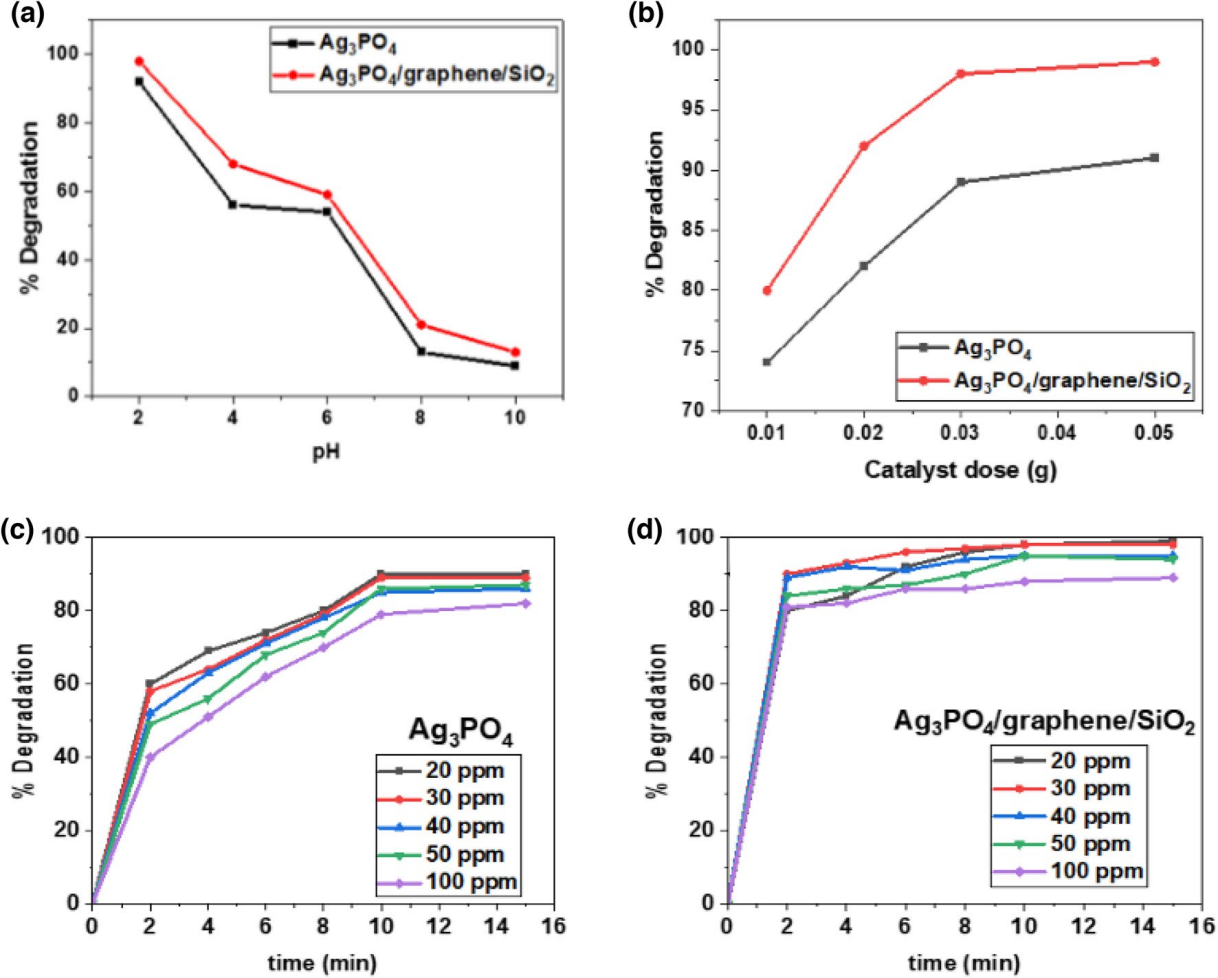
(**a**) pH, (**b**) catalyst dose, (**c**) and (**d**) time and initial concentration effects on the rate of degradation of TB using Ag_3_PO_4_ and Ag_3_PO_4_/G/SiO_2_.

The pH effect on photodegradation rate is shown in Fig. 7a, indicating the great effectiveness of both catalysts in the acidic medium, where the surfaces of the as-synthesized catalysts carry positive charges, which is consistent with the pHzpc investigation. According to the graph, at pH = 2, the percentage of dye degradation employing Ag_3_PO_4_ and Ag_3_PO_4_/G/SiO_2_ reached up to 89% and 98.5%, respectively. After that, they showed a gradual degradation decrease due to a decrease in the number of positive charges on catalyst surfaces. After the neutral medium, the degradation percent sharply decreased to approximately 10%, because the catalyst surfaces had become negatively charged according to pHzpc (Figure S1), resulting in generating an electrostatic repulsion force^25,34^.

#### Effect of catalyst dose

The effect of the catalyst dose was investigated to determine its effect on accelerating the degradation efficiency of the TB dye. As shown in Fig. 7b, as the catalyst dose increases, the percentage of TB degradation increases. This could be illustrated as by increasing the dose, sufficient surface area became available to serve the adsorption process, besides more radicals and positive holes will be generated which in turn serve the degradation rate^34,35^. The composite had a higher degradation efficiency than Ag_3_PO_4_, which was in line with BET analysis expectations because the composite had a higher specific surface area than Ag_3_PO_4_; thanks to the incorporated graphene/SiO_2_ composite which increased the adsorption area and improved the photocatalytic activity by trapping the excited electrons in the conduction band, therefore, minimizing their recombination with the valence band’s holes, which had a positive impact on the percent of degradation^36,37^. It is demonstrated from the graph that Ag_3_PO_4_ and Ag_3_PO_4_/G/SiO_2_ have a significant percentage of degradation at dose 0.03 g, with 89% and 98.5%, respectively. When the dose was increased up to 0.05 g the degradation percent was slightly increased. As a consequence, the appropriate used dose of each photocatalyst was 0.03 g.

#### Initial dye concentration and contact time effects

The effect of the initial dye concentration on the efficiency of photodegradation of TB dye was conducted at different initial concentrations (20, 30, 40, 50, 100 ppm) at constant operating conditions as shown in Fig. 7c,d. The results showed that as the initial concentration of the dye increases, the removal efficiency decreases^34^. For instance, for initial dye concentration 20 ppm, pH 2, and catalyst dosage 0.03 g the percent of degradation reached up to 89%, and 98.7% for Ag_3_PO_4_, and Ag_3_PO_4_/G/SiO_2_, respectively. As a consequence, the study emphasis the boosting effect of the composite in the degradation of TB dyes. Furthermore, the dye's degradation tendency revealed a rapid response in the first two minutes, but after careful analysis of the results, it appears that the composite is more active than pure Ag_3_PO_4_ in terms of degradation rate, followed by a slight degradation increase over the next four minutes, and finally a steady-state response. On the other hand, Ag_3_PO_4_ performed well in the first two minutes, but with a smaller percentage of degradation; after that, a progressive increase was detected for the next six minutes; eventually, the graph revealed a constant state for the final five minutes. The behavior of the photocatalysts is agreed with the BET results.

### Surface adsorption isotherm

In order to identify and study the behavior of the interaction between the dye and the adsorbent. Three adsorption isotherm models, which are Langmuir, Freundlich and Temkin, have been carried out. By analyzing the adsorption experimental data in dark medium, the most appropriate model will be utilized to investigate the kinetic model that the adsorption process follows^34,38–41^.

#### Langmuir isotherm

**Figure 8:**
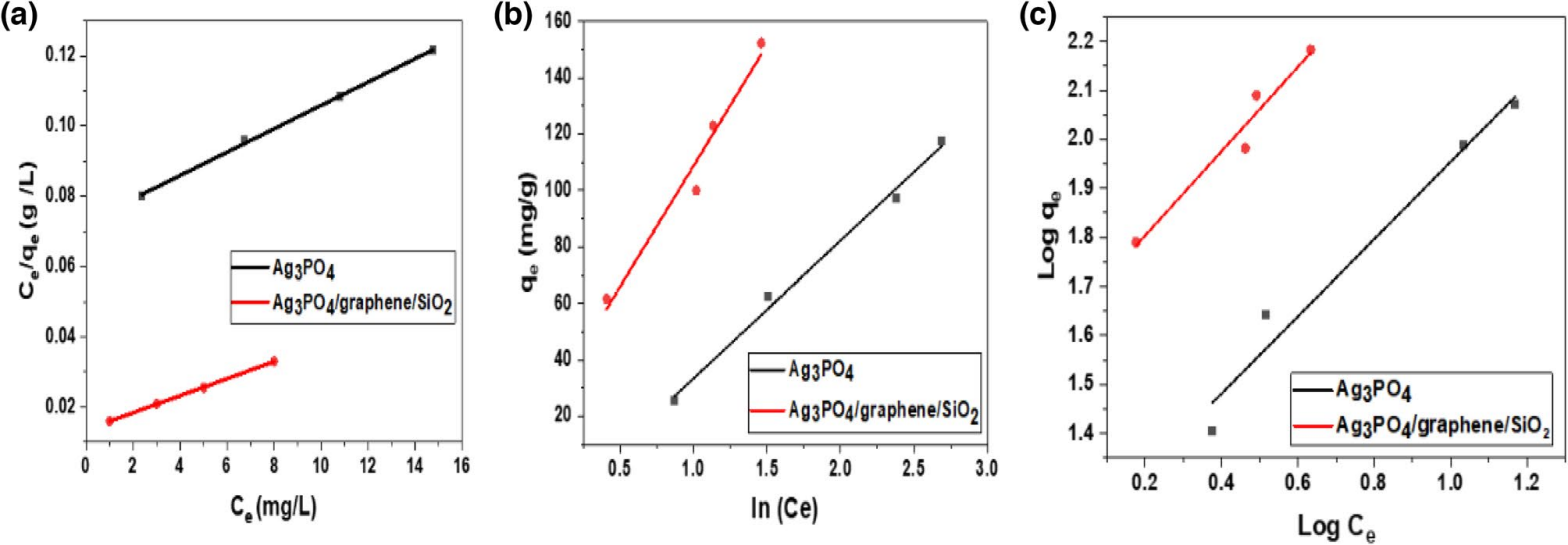
Adsorption isotherm models of TB on Ag_3_PO_4_ and Ag_3_PO_4_/G/SiO_2_. (**a**) Langmuir isotherm, (**b**) Freundlich isotherm and (**c**) Temkin isotherm.

The data was studied and analyzed using the Langmuir model, based on the linear formula of Langmuir Eq. (7). The separation factor (R_L_), a dimensionless constant, was also determined in order to express the isotherm's crucial properties using the following equation formula (8):

7 $$ \frac{{C_{e} }}{{q_{e} }} = \frac{1}{{K_{L} q_{max} }} + \frac{1}{{q_{max} }} $$

8 $$ R_{L} = \frac{1}{{1 + C_{i} K_{L} }} $$

where C_i_ and C_e_ are the adsorbate initial and equilibrium concentration (mg/l), respectively. q_e_ and q_max_ are the adsorption capacity adsorbed at equilibrium, and the maximum capacity (mg/g) respectively, and K_L_ is the Langmuir adsorption constant (l/mg). A plot of C_e_/q_e_ versus C_e_ was produced to determine q_max_ and K_L_ as shown in Fig. 8a and Table 3. When the R_L_ value is equal to zero, the adsorption is irreversible; when the value is between zero and one, the adsorption is favorable; when the value is larger than one. the adsorption is unfavorable; and when the value is equal to one, the adsorption is linear^42–44^. The calculated R_L_ values are shown in Table 3. All the values were dropped between zero and one which is favorable.

**Table 3:** The values of the separation factor R_L_ at different initial dye concentration using Ag_3_PO_4_, and Ag_3_PO_4_/G/SiO_2_.

Initial dye concentration (ppm)	R_L_	
	Ag_3_PO_4_	Ag_3_PO_4_/G/SiO_2_
20	0.5080	0.3052
30	0.4077	0.2265
40	0.3405	0.1801
50	0.2923	0.1494

#### Freundlich isotherm

The model that is built on heterogeneous adsorbent surfaces, was also used to experience the results. This isothermal model accomplished its hypothesis as a term that illustrates the heterogeneity of the surface and the exponential distribution of available sites. The linear formula of the isotherm is figured out in Eq. (9),^44^.

9 $$ Log q_{e} = Log K_{f} + \frac{1}{n} Log C_{e} $$

where K_f_ is adsorption capacity (L/mg), 1/n is adsorption intensity, C_e_ is the adsorbate equilibrium concentration (mg/L), q_e_ is the adsorption capacity adsorbed at equilibrium (mg/g). Figure 8b depicts a plot that expresses the relation between log q_e_ and log C_e_. A linear graph of Log (q_e_) against Log (C_e_) was drawn to detect the values of the slope (1/n) and intercept (Log K_f_) as shown in Fig. 8b and Table 4.

**Table 4:** The equilibrium parameter for the pre-mentioned three isothermal models.

Adsorbent	Langmuir constants			Freundlich constants			Temkin constants		
	q_max_	K_L_	R^2^	K_f_	n	R^2^	K_T_	B_T_	R^2^
Ag_3_PO_4_	282.486	0.0484	0.9997	15.076	1.2523	0.9553	0.7231	49.36	0.9903
Ag_3_PO_4_/G /SiO_2_	552.4861	0.1138	0.9964	42.806	1.1606	0.9372	1.3157	83.954	0.95

#### Temkin isotherm

This model assumes that as the concentration of adsorbate on the adsorbent surface increases, the heat of adsorption of all molecules in the layer decreases linearly due to interactions between them, and that the isothermal model is distinguished by symmetric distribution of binding energies up to maximum binding energy. Equation (10) presents the linear formula of Temkin isotherm.

10 $$ q_{e} = \frac{R}{{B_{T} }} ln K_{T} + \left( {\frac{R}{{B_{T} }}} \right)\ln C_{e} $$

where q_e_ is the amount adsorbed at equilibrium (mg/g), C_e_ is the adsorbate equilibrium concentration (mg/L), R is gas constant, K_T_ (L/mg) and BT are constants determined by graphing q_e_ against ln C_e_, as shown in Fig. 8c. Constants can be easily identified using the intercept and slope as shown in Fig. 8c and Table 4^37–40^.

The correlation coefficient of (R^2^) identifies the best appropriate model regarding the different equilibrium parameters for Langmuir, Freundlich, and Temkin isotherms for TB adsorption using Ag_3_PO_4_ and Ag_3_PO_4_ / G /SiO_2_.

From the previous data, q_max_ for the new composite is higher than that was calculated for graphene/SiO_2_ as it was 376 mg/g^34^, which ensure the greater adsorption capacity of Ag_3_PO_4_/graphene /SiO_2_. It can be concluded that the catalysts are more likely to follow the Langmuir isotherm model than other models which also revealed a well-fitting for the data. To sum up, the adsorption kinetic models fit the isothermal models in the following arrangement Langmuir > Temkin > Freundlich.

### photodegradation kinetic modeling

**Figure 9:**
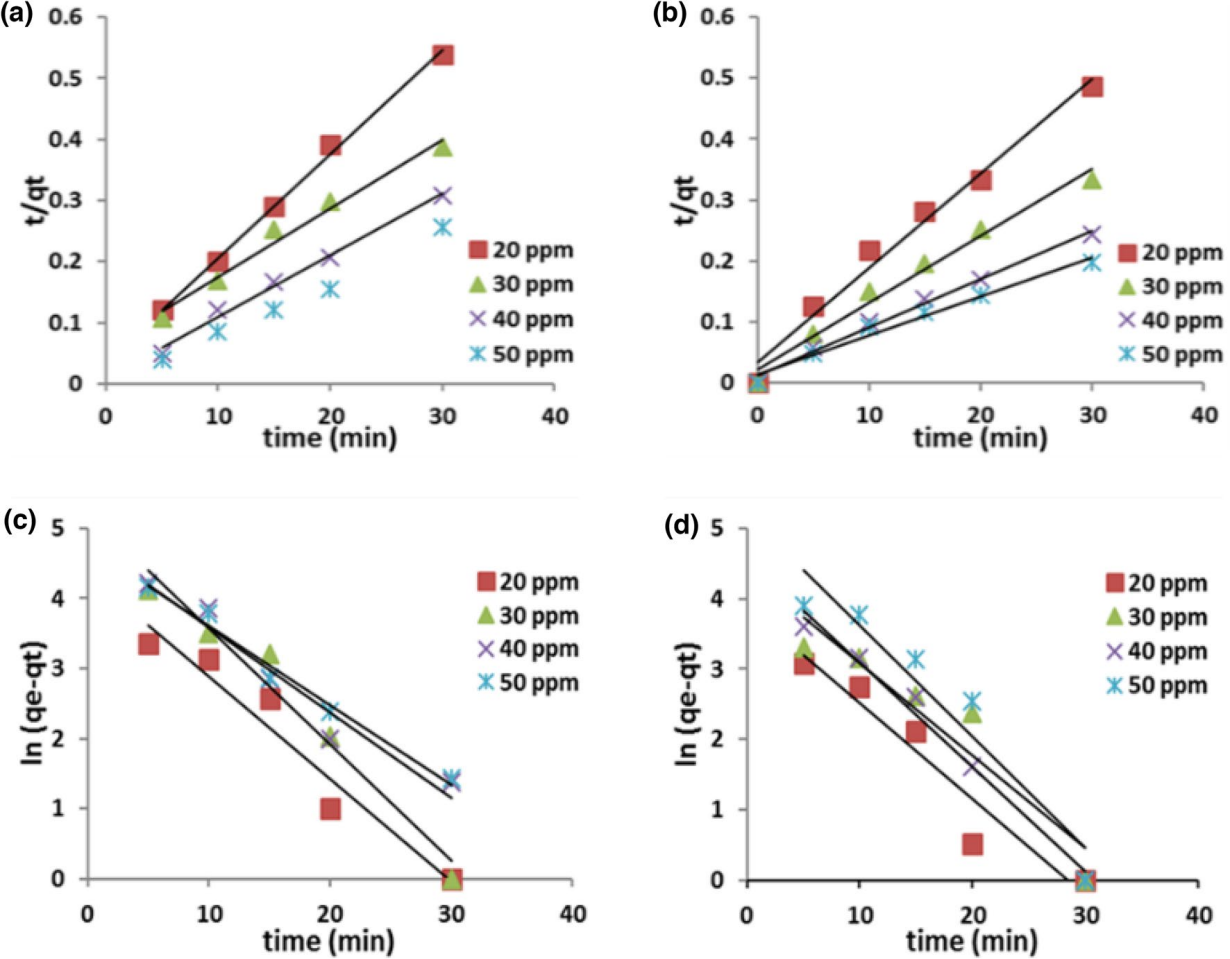
First order plots for various initial concentrations of TB removal using Ag_3_PO_4_ (**a**), Ag_3_PO_4_/G/SiO_2_ (**b**) and second order plots for various initial concentrations of TB removal using Ag_3_PO_4_ (**c**), Ag_3_PO_4_/G/SiO_2_ (**d**).

In order to study and investigate the mechanism of the photodegradation process, kinetic models have been employed. To explore the kinetics of the degradation process, pseudo first order and pseudo second order models have been constructed. Figure 9 shows the first and the second order models for TB elimination using Ag_3_PO_4_ and Ag_3_PO_4_/G/SiO_2_. A comparison of the experimental data was conducted in order to identify the model that was most appropriate for the obtained results. The optimal model was identified based on the degree of agreement between the computed and experimental values (q_e_), as well as the correlation coefficient R^2^^45–48^. Equations (11) and (12) show the linearized formulas for pseudo first and second order models, respectively.

11 $$ \ln \left( {q_{e} - q_{t} } \right) = lnq_{e} - K_{1} t $$

12 $$ \frac{t}{{q_{t} }} = \frac{1}{{K_{2} q_{e}^{2} }} + \frac{1}{{q_{e} }} $$

The experimental results for TB removal using Ag_3_PO_4_ and Ag_3_PO_4_/G/SiO_2_ are displayed in Tables 5 and 6, and proved that the photodegradation process fit more the second order kinetic model as the resultant experimental capacities were approximately equals to the calculated one. Moreover, the correlation factors R^2^ were also equal to 0.99 or higher which emphasis the convenience of the second-order.

**Table 5:** The pseudo first and second order kinetic parameters for TB removal using Ag_3_PO_4_.

Kinetic model		Pseudo 1st order			Pseudo 2nd order		
Initial conc (mg/g)	q_exp_ (mg/g)	K_1_ (min^−1^)	q_e_ (mg/g)	R^2^	K_2_ (g/mg.min)	q_e_ (mg/g)	R^2^
20	55.733	0.00487	77.3778	0.9429	0.00281	58.82353	0.9967
30	77.5	0.00453	186.1588	0.9634	0.00256	78.74016	0.9812
40	97.3	0.00406	122.0584	0.9505	0.00116	99.0099	0.9923
50	117.5	0.00377	113.7838	0.9833	0.00089	117.6471	0.9923

**Table 6:** The pseudo first and second order kinetic parameters for TB removal using Ag_3_PO_4_/G/SiO_2_.

Kinetic model		Pseudo 1st order			Pseudo 2nd order		
Initial conc (mg/g)	q _exp_ (mg/g)	K_1_ (min^−1^)	q_e_ (mg/g)	R^2^	K_2_ (g/mg.min)	q_e_ (mg/g)	R^2^
20	61.667	0.00454	48.03837	0.9187	0.00891	64.51613	0.9915
30	90.333	0.00496	76.63623	0.8873	0.00535	91.74312	0.9909
40	123	0.00442	95.08774	0.9835	0.00408	126.5823	0.9908
50	152.333	0.00402	95.08774	0.916	0.00204	156.25	0.99

From the previous data shown in Tables 3, and 4, it could be concluded that the nanocomposite showed higher reaction rate constants (K_2_). The average of the rate constants increased by 2.53 times that of Ag_3_PO_4_. This is can be explained as the composite possess a larger surface area and better-regulated morphology with well particles distribution; therefore, the degradation efficiency is promoted. BET, SEM, and TEM analyses corroborate these findings^26^.

### Proposed photodegradation mechanism

When the silver phosphate is irradiated with visible light, electrons in the material are excited to a higher energy level, creating electron–hole pairs. The electron–hole pairs thus created are separated due to the presence of G/SiO_2_ nanocomposite, which traps the photo-excited electrons. The excited electrons can reduce the atmospheric oxygen to O_**2**_^.^ radical which could lead to the breakdown of pollutants to carbon dioxide and water, and the holes present in the valance band will react with water molecules and generate hydroxyl radicals (OH^.^) that are very strong oxidizing agents, which helps in the degradation of TB dye^41,42^. Also, the adsorption of oxygen onto the G/SiO_2_ surface will continuously oxidize the TB dye and release carbon dioxide and water. The regenerated electrons can then recombine with the holes trapped in the bulk of the Ag_3_PO_4_/G/SiO_2_ nanocomposites to produce more excited electron–hole pairs, which can repeat the photocatalytic process as shown in Fig. 1. The scavenger experiments were conducted to determine the key active species (h^+^, e, ^·^OH and ^·^O_2_) involved during the photocatalytic process. As seen in Figure S5, the significant reduction was observed on addition of propanol, suggesting that ^·^OH was the dominant species during the degradation of dyes. The addition of oxalic acid (h^+^ and methanol (^·^O_2_) scavengers resulted in only slight reduction of photo- activity. The order of photocatalytic suppression after addition of different scavengers were ^·^OH > e^−^ > h^+^>^·^O_2_.

In brief, the Ag_3_PO_4_/G/SiO_2_ composites act as a visible light photocatalyst for the degradation of organic pollutants, and G/SiO_2_ playing a major role by acting as a support to promote dye adsorption, an electron acceptor and also delaying the recombination rate of electron–hole pairs, which are responsible for the photocatalytic reactions^43^.

### Characterization of the used catalyst

Some characteristic analyses were performed to the used photocatalysts in order to inspect their morphology and status, seeking to proof that, all the adsorbed dye molecules on the catalyst surface were degraded after light illumination which in turn boost the efficiency of both photocatalysts. FTIR, EDS, and SEM–EDS mapping tests are shown below. Where Fig. S2, FTIR analysis of the used catalysts after they were centrifuged at 6000 rpm and dried for 12 h in a vacuum oven at 60 °C to ensure the degradation of TB dye and the absence of adsorbed molecules on the catalyst surface. It was predicted that the FTIR result would reveal the same functional groups for each catalyst as mentioned before. SEM–EDS mapping results are shown in Fig. S3 and Fig. S4, from these results, we can conduct the coexistence of both catalysts’ elements and no elements of the dye were present which is compatible with the FTIR results. These results ensure the degradation of the TB dye.

A comprehensive study has been done to investigate and compare the synthesized catalysts' activity and efficiency in comparison with other photocatalysis used for the degradation of trypan blue as an organic pollutant. Table 7 shows the comparison with respect to TB as a pollutant source.

**Table 7:** A comparison of photocatalytic degradation efficiency using various catalysts, in terms of TB degradation.

#	Dye	Photocatalyst	Conditions	Removal (%)	References
1	TB	Se doped ZnO NPs	pH 5 for 6 h, dose 0.6 mg/ml, 2 h,30w power UV light	89.1 ± 3.1	^49^
2	TB	6 wt% BaF_2_–TiO_2_	TB = 1 × 10 − 4 M, catalyst = 4 g L − 1, pH = 6.2, 30 min	96.30	^50^
3	TB	Ag_3_PO_4_/Bi_2_S_3_	Ci = 25 ppm, 25 min of irradiation and sonication time, pH 6.0, and 0.25 g/L of photocatalyst dosage	98.44	^51^
4	TB	TiO_2_	1gm/L of TiO_2_ at pH = 6, T = 293 K after 75 min, Ci = 3*10^-5 M	46	^52^
5	TB	ZnO/Ag	120 min,25 μg/L,100 ml, 0.1 g dose, pH 10	80	^53^
6	TB	SnS nanorods capped with mercapto acetic acid	dye degradation in 4 h,1 × 10 − 5 M, sunlight for 5 h	95	^54^
7	TB	Ag_2_C_2_O_4_/Ag/g-C_3_N_4_	30 min, pH = 6, Ci = 5 ppm, dose = 0.015 g, Booster mirror reactor	85.90	^55^
8	TB	Ag_3_PO_4_	100 ml, 0.03 g dose, pH 2, illumination time 30 min, Ci = 30 ppm	92	Present study
9	TB	Ag_3_PO_4_/graphene/SiO_2_	100 ml, 0.03 g dose, pH 2, illumination time 20 min, Ci = 30 ppm	98	Present study

## Conclusions

The novel Ag_3_PO_4_/G/SiO_2_ nanocomposite has been successfully synthesized by co-precipitating of Ag_3_PO_4_ with pre-synthesized green graphene/SiO_2_ nanocomposite. The characterization results ensure the formation of the composite. Moreover, the characterization results manifested the improvement of the Ag_3_PO_4_ photocatalyst by increasing its active surface area and optical properties, where the band gap energy of the Ag_3_PO_4_/G/SiO_2_ nanocomposite was 2.33 eV, and for Ag_3_PO_4_ was 2.4 eV. The photodegradation of TB showed better efficiency after using 0.03 g from both catalysts at initial concentration of 30 ppm. The percent of degradation reached 89%, and 98.7% by using Ag_3_PO_4_ and Ag_3_PO_4_/G/SiO_2_, respectively. Hence, the rate of the photo degradation of the dye become faster and the removal efficiency increased. Based on the adsorption isotherm analysis, Langmuir model is the most suitable isothermal model, which means that the Adsorption is proportional to the percentage of the surface of an adsorbent that is available. Kinetic study showed that the degradation process on both catalyst surfaces follows pseudo second order. The future prospectives for this work is to study the effect of addition different concentration of graphene/ SiO_2_ nanocomposite on different photocatalysts rather than silver phosphate.

### Supplementary Information

Supplementary Figures.

## Data Availability

The datasets used and/or analyzed during the current study available from the corresponding author on reasonable request.
