# Supplementary material for: Formulation of silver phosphate/graphene/silica nanocomposite for enhancing the photocatalytic degradation of trypan blue dye in aqueous solution
Source: Sci Rep. 2024 Jul 10;14:15885. doi: 10.1038/s41598-024-66054-5 (PMC11237074; doi:10.1038/s41598-024-66054-5)
Supplement: Supplementary file 1 — Supplementary Figures. [file 41598_2024_66054_MOESM1_ESM.docx]

Fig. S1. Determination of the zero point charge of Ag_3_PO_4_, and Ag_3_PO_4_ /graphene/SiO_2_ (pHzpc)

**Characterization of the used catalyst**

some characteristic analyses were performed to the used photocatalysts in order to inspect their morphology and status, seeking to proof that, all the adsorbed dye molecules on the catalyst surface were degraded after light illumination which in turn boost the efficiency of both photocatalysts. FTIR, EDS, and SEM-EDS mapping tests are shown below. Where fig S2, FTIR analysis of the used catalysts after they were centrifuged at 6000 rpm and dried for 12 hours in a vacuum oven at 60°C to ensure the degradation of TB dye and the absence of adsorbed molecules on the catalyst surface. it was predicted that the FTIR result would reveal the same functional groups for each catalyst as mentioned before.

Fig. S2 FTIR of used Ag_3_PO_4_ (a), and Ag_3_PO_4_/graphene/SiO_2_ composite (b)

EDS and SEM-EDS mapping results are shown in fig S3 and fig S4, from these results, we can conduct the coexistence of both catalysts’ elements and no elements of the dye were present which is compatible with the FTIR results. These results ensure the degradation of the TB dye.


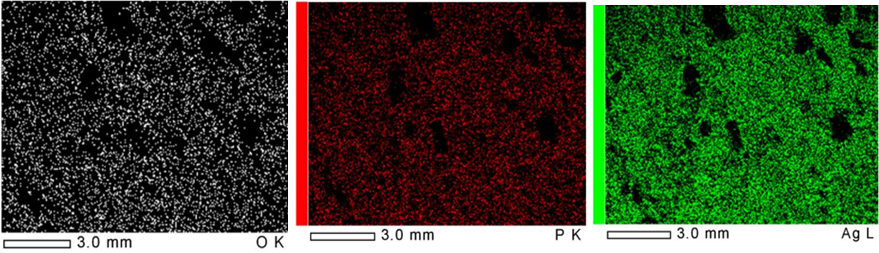


**a**


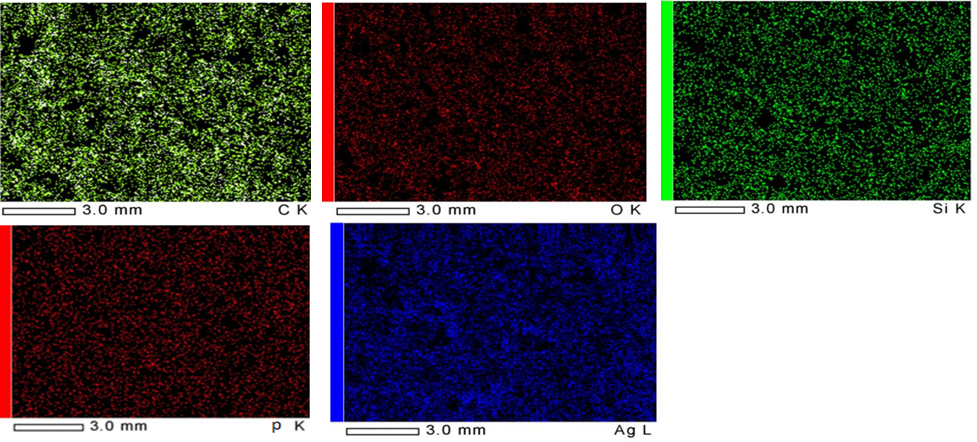


**b**

Figure S3. SEM-EDS Mapping Images for Ag_3_PO_4_(a), and Ag3PO4/graphene/SiO_2_ (b)

Figure S4. EDS analysis of used Ag_3_PO_4_ and Ag_3_PO_4_/graphene/SiO_2_

Figure S5. Different active species scavengers for the degradation of tryban blue dye using 0.03 gm of Ag_3_PO_4_/graphene/SiO_2_ photo catalyst
